# Costs and Cost-Effectiveness of Training Traditional Birth Attendants to Reduce Neonatal Mortality in the Lufwanyama Neonatal Survival Study (LUNESP)

**DOI:** 10.1371/journal.pone.0035560

**Published:** 2012-04-24

**Authors:** Lora L. Sabin, Anna B. Knapp, William B. MacLeod, Grace Phiri-Mazala, Joshua Kasimba, Davidson H. Hamer, Christopher J. Gill

**Affiliations:** 1 Center for Global Health and Development, Boston University, Boston, Massachusetts, United States of America; 2 Department of International Health, Boston University School of Public Health, Boston, Massachusetts, United States of America; 3 Lufwanyama District Health Management Team, Kalulushi, Zambia; 4 Zambia Centre for Applied Health Research and Development, Lusaka, Zambia; 5 Infectious Diseases Section, Department of Medicine, Boston University School of Medicine, Boston, Massachusetts, United States of America; Vanderbilt University, United States of America

## Abstract

**Background:**

The Lufwanyama Neonatal Survival Project (“LUNESP”) was a cluster randomized, controlled trial that showed that training traditional birth attendants (TBAs) to perform interventions targeting birth asphyxia, hypothermia, and neonatal sepsis reduced all-cause neonatal mortality by 45%. This companion analysis was undertaken to analyze intervention costs and cost-effectiveness, and factors that might improve cost-effectiveness.

**Methods and Findings:**

We calculated LUNESP's financial and economic costs and the economic cost of implementation for a forecasted ten-year program (2011–2020). In each case, we calculated the incremental cost per death avoided and disability-adjusted life years (DALYs) averted in real 2011 US dollars. The forecasted 10-year program analysis included a base case as well as ‘conservative’ and ‘optimistic’ scenarios. Uncertainty was characterized using one-way sensitivity analyses and a multivariate probabilistic sensitivity analysis. The estimated financial and economic costs of LUNESP were $118,574 and $127,756, respectively, or $49,469 and $53,550 per year. Fixed costs accounted for nearly 90% of total costs. For the 10-year program, discounted total and annual program costs were $256,455 and $26,834 respectively; for the base case, optimistic, and conservative scenarios, the estimated cost per death avoided was $1,866, $591, and $3,024, and cost per DALY averted was $74, $24, and $120, respectively. Outcomes were robust to variations in local costs, but sensitive to variations in intervention effect size, number of births attended by TBAs, and the extent of foreign consultants' participation.

**Conclusions:**

Based on established guidelines, the strategy of using trained TBAs to reduce neonatal mortality was ‘highly cost effective’. We strongly recommend consideration of this approach for other remote rural populations with limited access to health care.

## Introduction

In many low-resource countries, neonatal mortality contributes approximately 40% of all under five-year mortality, with birth asphyxia and neonatal sepsis constituting the majority of preventable neonatal deaths [1], [2]. A major factor in high neonatal mortality is the high proportion of home births. It is estimated that 60 million deliveries occur each year outside of health facilities [3]; in many low-resource areas over 50% of all births occur in the community, largely in mothers' homes [4]. In many areas, traditional birth attendants (TBAs) are an essential source of basic obstetrical care. TBAs have proven effective in a variety of secondary roles in the community, such as serving as peer educators or breast feeding counselors, but their proximity to the mother/infant pair and their location within the community suggests that TBAs could play a more direct role in reducing neonatal deaths as well.

To test the hypothesis that TBAs could effectively reduce neonatal mortality in a rural African setting, we conducted a randomized controlled effectiveness study in Zambia called the Lufwanyama Neonatal Survival Project (LUNESP) [5]. In LUNESP, TBAs were randomized to receive training and equipment to allow them to perform a set of interventions targeting several of the main causes of neonatal mortality. The interventions included two components: 1) Neonatal Resuscitation Protocol (NRP), which aimed to reduce deaths due to birth asphyxia and hypothermia; and 2) antibiotics with facilitated referral to a health center (AFR), which aimed to reduce sepsis deaths during the first month of life. The primary endpoint was a comparison of mortality rates by day 28 of life among live-born infants. Based on outcomes for 3355 deliveries, we found that infants delivered by intervention TBAs were 45% less likely to die than infants delivered by control TBAs (RR 0.55, 95% CI 0.33 to 0.90). This equated to one death avoided for every 56 deliveries attended by an intervention TBA, for an absolute reduction of 18 deaths per 1000 live births [5].

Here we report on the economic evaluation component of the LUNESP project. We present our findings from several perspectives. First, we present a financial analysis, based on the actual costs incurred during the intervention. Second, we present an economic analysis, which utilized a societal perspective of costs. Lastly, as evidence for policy-makers, we present a forecasted economic analysis in which the costs and cost-effectiveness of a modified model of the intervention are projected over a ten-year timeframe.

## Methods

### Study summary

LUNESP was a cluster-randomized trial conducted from 2006–2008 in an impoverished rural district in north-central Zambia among a population with limited access to health care [5]. A total of 120 TBAs were initially randomized to receive the intervention trainings and equipment, or to continue their existing standard of care. All TBAs received one ‘clean delivery’ kit per birth for their regular TBA duties, which included a delivery sheet, cord cutter, cotton cord ties, latex gloves, and soap. Training for intervention TBAs commenced with 4-day sessions for each group of 30 TBAs, followed by 1–2 day refresher trainings approximately every 3–4 months for the duration of the trial. The trainings were conducted collaboratively by a US-based neonatologist and a local master trainer (a Zambian nurse-midwife), assisted by 6–8 Zambian facilitators. To demonstrate competency, intervention TBAs indicated skills retention at each retraining session. Each intervention TBA received one resuscitator mask, a polypropylene bottle with chlorinated water, and a laminated reference card summarizing NRP and trigger conditions for AFR. They also received each of the following per delivery: two flannel receiving blankets, a soft rubber bulb syringe, two 250 mg amoxicillin capsules, one 2-ounce mixing cup and spoon, and a 3 ml oral syringe. A more detailed description of the design, training, and analytic methods used in LUNESP has been published elsewhere [5].

The study was approved by the ethical committees at Boston University Medical Center (Boston, MA) and the Tropical Diseases Research Centre (Ndola, Zambia). All TBAs and mothers who participated in the LUNESP trial provided written informed consent, using forms in English and the local languages Bemba and Lamba. We did not obtain separate informed consent from participants for the present analysis because we utilized de-identified, aggregated outcome data only from the trial. The LUNESP trial was registered as clinicaltrials.gov NCT00518856.

### Cost analyses

Analyses were conducted from three perspectives: 1) a financial analysis based on costs incurred during LUNESP; 2) an economic analysis, which factored in societal costs; and 3) a forecasted 10-year economic analysis, which modeled expected intervention economic costs and effectiveness over a future program-appropriate timeframe in order to provide practical information for policy-makers beyond data generated by a short-term research study alone.

#### Financial analysis

The financial analysis was based on the incremental expenditures related to the 33-month intervention. This encompassed 6 months of start-up activities in February–July 2006, including the first training and 27 months of implementation from August 2006-October 2008) (Table 1). All research- and control group-related costs were excluded. We included personnel time for: start-up (US-and Zambia-based collaborators); program monitoring and supervision (Zambian project staff); project coordination (Zambian project staff); and training. We included the costs of travel, food, and accommodation associated with training. Some travel expenses by the US-based neonatologist were defrayed due to cost-sharing with other projects; these savings were incorporated. TBAs were not paid by the government or the study and thus no salaries were included.

**Table 1:** Items included in cost analysis.

Personnel Costs	Comments
Personnel costs during start up activities	30 days preparation time each for the US and Zambian teams
Personnel costs for monitoring during the interventions	Zambian project director: 2 days/month
Personnel costs for training workshops	Zambian TBA trainer: 5 days/month
	Training facilitators (6 facilitators)
	Project coordinator: 4 days/month
	US neonatologist attending trainings: attended 5 of 9 workshops^1^
	Zambian trainers attended 9 of 9 workshops
Travel, food and lodging for TBAs attending workshops	9 workshops×60 intervention TBAs

Supply Costs	Comments
Training supplies	Infant resuscitation mannequins, printed materials
Intervention supplies – one-time costs (per TBA)	Resuscitation mask; polypropylene water bottle used to make slurry with amoxicillin); medication cup; mixing spoon; laminated card pictorially summarizing steps of NRP (front) and AFR (back)
Intervention supplies – recurrent costs (per delivery by an intervention TBA)	1 safe delivery kit, 1 bulb syringe, 2 flannel receiving blankets, 2 amoxicillin tablets (250 mg), oral syringe (for administering amoxicillin/water slurry)

1 Some travel expenses of the US-based neonatologist were defrayed due to cost-sharing related to airline tickets and local food and accommodation. In the financial analysis, costs incorporated these savings.

Supply costs included training items (resuscitation mannequins, printing, and stationary); single items provided to each TBA at the beginning of the intervention such as a mask, instruction card, and a mixing spoon; and items needed for each birth, including blankets and bulb syringes. Some supplies were purchased locally and others were purchased in the US and brought to Zambia by project staff. We included a one-time customs charge applied to supplies carried into Zambia and a shipping fee for items shipped from the US in 2006. The District Health Management Team (DHMT) was responsible for providing amoxicillin and clean delivery kits, though due to occasional supply issues, the program made several purchases of both items, the costs of which were included. Nominal Zambia-based costs were converted to US dollar values using the average annual exchange rate for the year in which the costs were incurred [6] and then added to nominal US dollar-based costs. We adjusted total annual US dollar costs by US inflation rates (Consumer Price Index) [7] and expressed total program costs in real 2006 US dollar figures, categorized as fixed and variable costs over the life of the project. We also estimated the 2006 Present Value (PV) of financial costs (using a discount rate of 3%) [8], and the 2011 PV for greater comparability with additional analyses.

#### Economic analysis

As recommended by the World Health Organization, we utilized a societal perspective for the economic analysis to better capture the economic resource cost of intervention inputs [9]. First, we included payment to TBAs for their participation. This was based on the supply price of TBAs, estimated by calculating the difference in reported average cash payments per birth made by families to intervention TBAs vs. control TBAs. Second, we eliminated cost-sharing of expenses (i.e., subsidized travel of US-based neonatologist, donations of key supplies). Third, we included the cost of amoxicillin tablets throughout the project, using an estimated international price of US$ 0.1 per 250 mg dose (but assumed consistent delivery kits by the DHMT per their mandate) [10]. Fourth, we deducted the customs tax payment, since taxes are an economic transfer and not an actual resource cost. All US dollar nominal costs were adjusted for US inflation rates [7], discounted at 3%, and expressed in 2011 US dollar PV terms.

#### Forecasted 10-year analysis

We utilized a societal perspective, with a ten-year program lifespan from January 2011 through 2020. We assumed a similar model as that of the trial: 6 months of start-up, 60 participating TBAs, initial intensive 4-day training followed by three 2-day refresher trainings per year, the same supply inputs, and similar levels of local personnel participation in training and monitoring and supervision activities. However, we made several adjustments to the model to localize the intervention and increase its long-term sustainability. We assumed task-shifting by replacing the US-based neonatologist with a local skilled nurse-midwife who would have 5 full-time facilitators for assistance with trainings. We anticipated that TBA turnover would be minimal over time, with training of new TBAs managed by including them in ongoing refresher training rather than holding additional new trainings. US program management was replaced with a team of 3 local staff who would establish the intervention over 30 days. We also estimated compensation for TBAs on the basis of the per birth payments made by families to intervention TBAs during the trial. All nominal costs included expected inflation [7], [11]; nominal TBA compensation also incorporated a real annual increase of 1%. We assumed no improvements in or loss of efficiency over time, resulting in similar annual costs after the start-up period. Annual Zambia-based costs in local currency were converted to nominal US dollars using an expected constant real exchange rate equal to the 2011 rate [6] and added to annual US dollar costs. Total annual costs were deflated by expected inflation and discounted (at 3%) to estimate the 2011 PV.

### Cost-effectiveness analyses

We estimated the cost effectiveness of the LUNESP interventions using the formula: ICEA = (C_I_−C_C_)/(M_I_−M_C_), where ICEA is the incremental cost-effectiveness ratio; C_I_ and C_C_ are the total (discounted) costs related to the intervention and control groups, respectively; and M_I_−M_C_ are the (discounted) mortality figures for intervention and control groups, respectively. Because all costs were incremental, C_C_ = 0. The difference in mortality per year, (M_I_−M_C_)_t_, where t = years 2006, 2007, 2008, was the difference in measured mortality rates per 1,000 live births between intervention and control groups in each project year. Annual lives saved were discounted at 3% per year. We also estimated the number of averted disability-adjusted life years (DALYs), using an approach that incorporates expected years of life lost (not country-specific), with time lived at different ages valued using an exponential function and discounted at 3% [12].

For the forecasted CEA we conducted three analyses. First, our base case used the estimated economic costs for the 10-year program and assumed that reduced mortality would equal the trial's overall effect size (17.9/1000 births). The number of expected births per TBA was defined as the annual number observed in 2008 (when TBA activity was at its peak) (1.29 births per TBA per month). We estimated both undiscounted and discounted (at 3%) annual deaths avoided, though used the latter to calculate the incremental economic cost per death avoided and DALY averted. In addition, we examined the cost-effectiveness of two alternative ‘optimistic’ and ‘conservative’ scenarios. The former retained the trial's mortality effect and assumed program implementation under more stringent conditions by increasing the number of TBAs per cohort trained from 60 to 80, reducing the refresher training time from 2 to 1 days, reducing monitoring activities from a monthly to bi-monthly timetable, and assuming births based on Zambia's average national density rather than sparsely-populated Lufwanyama (see Table 2). The conservative scenario, relative to the base case, involved reducing the effect size by 25% to 13.4/1000 births, decreasing the mean monthly number of births per TBA to the overall average observed in the trial (1.21), and increasing the number of annual refresher trainings from 3 to 4.

**Table 2:** Input values for multivariate sensitivity analysis, by scenario.

Parameter	Type of probability distribution^1^	Minimum Value^2^	Likeliest value	Maximum Value^2^	Sources
**Uncertain variables**					
Mortality effect					
Base case	Triangular	4.1	17.9	31.8	LUNESP trial
Optimistic scenario	Triangular	4.1	17.9	31.8	LUNESP trial
Conservative scenario	Triangular	3.1	13.4	23.9	Estimate^3^
Births attended per BA/month					
Base case	Triangular	0.70	1.29	1.87	LUNESP trial^4^
Optimistic scenario	Triangular	1.82	3.34	4.86	Estimate^5^
Conservative scenario	Triangular	0.49	1.21	1.93	LUNESP trial^6^
Cost of food/day/participant during training workshops^7^					
Base case	Triangular	10.6	14.1	17.7	LUNESP trial
Optimistic scenario	Triangular	10.6	14.1	17.7	LUNESP trial
Conservative scenario	Triangular	10.6	14.1	17.7	LUNESP trial
Cost of fuel, car, staff for program monitoring/month^7^					
Base case	Triangular	466	622	777	LUNESP trial
Optimistic scenario	Triangular	466	622	777	LUNESP trial
Conservative scenario	Triangular	466	622	777	LUNESP trial
**Policy-determined variables**					
Number of trainings/year					
Base case			3		LUNESP trial
Optimistic scenario			2		Estimate^8^
Conservative scenario			4		Estimate^8^
Number of days/training					
Base case			2		LUNESP trial
Optimistic scenario			1		Estimate^8^
Conservative scenario			2		LUNESP trial
Months/year of monitoring					
Base case			12		LUNESP trial
Optimistic scenario			6		Estimate^8^
Conservative scenario			12		LUNESP trial
Number of TBAs					
Base case			60		LUNESP trial
Optimistic scenario			80		Estimate^8^
Conservative scenario			60		LUNESP trial

1 Triangular distribution was chosen to be consistent with the approach used in other cost-effectiveness studies [24]–[26].

2 For parameters except births/TBA, these are minimum and maximum values. For births/TBA, these are 5% and 95% values determined by the 95% CI estimated from the standard deviation of monthly mean births/TBA from the LUNESP trial.

3 25% less than the LUNESP trial's mortality effect. Using a +/−25% range is typical in similar studies [17].

4 Based on the average number of births attended/TBA/month during the final year of the LUNESP trial.

5 Estimated value for typical area in Zambia, calculated by multiplying the LUNESP trial's mean value by the factor: (Zambia national population density/average population density in Lufwanyama).

6 Based on the average number of births attended/TBA/month during the 27 months of LUNESP trial implementation.

7 Costs in estimated 2011 US$.

8 Authors' estimates based on experience during the LUNESP trial.

Interpretations regarding ‘cost-effectiveness’ were based on the 2001 recommendation of the Commission on Macroeconomics and Health and adopted by the World Health Organization (WHO), whereby an intervention is ‘highly cost-effective’ if it averts a DALY for less than per capita GDP (Gross Domestic Product) and ‘cost-effective’ if it averts a DALY for less than 3 times per capita GDP [13].

### Sensitivity analyses

For the base case, we conducted univariate sensitivity analyses to explore the impact on the costs and cost effectiveness of the 10-year program of varying key input parameters. Certain variables were uncertain—mortality effects, number of births per TBA, and input costs. Others were pre-determined, either wholly or in part, by program or policy staff, including the number of refresher training workshops per year, number of days of each workshop, degree of involvement of the US-based neonatologist in training activities, and number of participating TBAs trained per workshop.

We also conducted multivariate sensitivity analysis with Monte-Carlo simulations using Crystal Ball™ (Oracle Corp, Redwood Shores, CA) to test the model's robustness for each of the three forecasted scenarios given simultaneous changes in key parameters, an approach commonly employed in cost-effectiveness analyses [14]–[16]. Based on the results of the one-way sensitivity analysis for the base case, we selected the four uncertain input parameters (e.g., non-policy determined) whose variations had the greatest impact on the cost-effectiveness outcomes to include in this analysis: intervention effect size, average deliveries/month/TBA, logistic costs of trainings (e.g., room and board), and cost of travel for monitoring and supervision. The probability distributions for input parameters were assumed to be triangular. For the mortality effect, the likeliest value was taken from the trial, with minimum and maximum ranges set at the trial's 95% confidence interval (CI). For the number of births per TBA, we used the trial mean and standard deviation to estimate a 95% CI. For 2011 cost parameters, we used the 2011 estimated costs for likeliest values with minimum and maximum ranges of ±25% (Table 2), an approach frequently employed in similar cost studies [17].

## Results

### Intervention costs and cost-effectiveness

In the financial analysis, the program intervention costs in real 2006 US$ were $47,020, $32,599, and $29,282 in 2006, 2007, and 2008, respectively (Table 3). After discounting, the 2006 PV was $106,271. The cost of start-up and supplies, as well as the longer training workshop in June 2006, was primarily responsible for the higher annual costs in 2006 relative to 2007–08. The cost difference between 2007 and 2008 reflected the fact that the study was implemented for only 10 months in 2008. Approximately two-thirds of the cost of the program's first year was US-based, whereas in 2007 and 2008, US-based costs represented less than 30% of total costs. Twenty-seven percent of total financial costs were for the US-based neonatologist.

**Table 3:** Summary of the financial costs of the LUNESP intervention: 2006–08.

Parameters		2006	2007	2008
Number of months of intervention set-up		6		
Number of months of intervention implementation		5	12	10
**A. Zambia in-country costs (US$)** 1				
Personnel salaries				
Program set-up and monitoring		3129	11321	9434
Training: trainers/facilitators		3442	9794	8978
Food & accommodation (trainings)				
Trainers/facilitators		919	7414	7626
TBAs^2^		3597	20269	20032
Travel				
Program set-up and monitoring		1123	19013	22217
TBAs: trainings^2^		3450	19160	10261
Supplies				
Infant receiving blankets		1406	11500	5000
Amoxicillin		107	80	0
Clean delivery kits		0	1542	546
Printing & stationary – trainings		267	1139	2891
Miscellaneous				
Customs clearance charge		281		
**Total Zambia in-country costs**		**17721**	**24608**	**22785**
**B. US-based costs (US$)**				
Personnel salaries				
Program set-up and monitoring		4961	0	0
Training: Neonatologist		9244	6409	2499
Food & Accommodation (trainings)				
Neonatologist		0	0	132
Travel (US-Zambia for trainings)				
Neonatologist		4370	2511	4441
Supplies				
Infant receiving blankets		1426	0	0
Laederal resuscitation masks		1095	0	0
Resuscibaby manikins		2125	0	0
Laminated instruction cards		112	0	0
Bulb syringes		4320	0	1190
Plastic spoons		65	0	0
Miscellaneous				
Shipping: supplies to Zambia		1583		
**Total US-based costs**		**29299**	**8920**	**8488**
**Total US-based & Zambia in-country costs**		**47020**	**33528**	**31273**
**Total Costs: Real 2006 US$**		**47020**	**32599**	**29282**
***Present Value: 2006 US$*** 3	***$106271***			

1 In-country costs were converted to US dollar values at the following average annual ZMK/US$ exchange rates: 3557 (2006), 4114 (2007), and 3818 (2008) [6].

2 60 TBAs.

3 Discount rate = 3%.

LUNESP's total economic cost was 8% higher than the total financial cost, mainly due to costs associated with the US-based neonatologist's attendance at all workshops (Table 4). Fixed costs accounted for nearly 90% of economic costs, among which the largest component was training activities (63–65% of total annual costs). Thirty-one percent of all economic costs were for the US-based neonatologist.

**Table 4:** Fixed and variable costs of the LUNESP interventions (2011 US$)^1^

Cost item	Financial		Economic		Projected Economic	
	2006–08		2006–08		2011–20	
	Cost % total		Cost % total		Cost % total	
**Total fixed costs** 2	104514	88.1	111902	87.6	208306	81.2
Program set-up	7268	6.1	7268	5.7	1531	0.6
Monitoring & supervision	19256	16.2	19256	15.1	83092	32.0
Training workshops	74806	63.1	82507	64.6	120063	46.8
Foreign personnel^3^	31771	26.8	39473	30.9	-	0.0
Local personnel^4^	11036	9.3	11036	8.6	28686	11.2
TBAs^5^	25773	21.7	25773	20.2	85079	33.2
Supplies^6^	3706	3.1	3706	2.9	6298	2.5
Supplies (non-training)^7^	1418	1.2	1418	1.1	2185	0.9
**Total variable costs** 2	14060	11.9	15854	12.4	48149	18.8
Supplies^8^	14060	11.9	14353	11.2	32150	12.5
TBA compensation^9^	-	0.0	1501	1.2	16000	6.2
**Total costs**	**118574**	**100.0**	**127756**	**100.0**	**256455**	**100.0**
Cost per program year^10^	49469		53550		26834	
Cost per birth^11^	62.8		67.6		29.1	
Variable costs per birth	7.4		8.4		5.3	

1 All figures are in discounted (at 3%) real 2011 US$.

2 Fixed costs include program set-up, monitoring, training, and supplies provided to TBAs on a per-TBA basis; variable costs include supplies provided on a per birth basis and compensation toTBAs), since these are estimated based on average deliveries/month.

3 Participation of one US-based neonatologist in training workshops, including time spent (salary), travel, and in-country food/accommodation.

4 2006–08 intervention: assistant trainer, program director, local supervisor, and 4 additional full-time facilitators during trainings. 2011–2020 intervention: lead trainer (nurse midwife) and five facilitators.

5 60 TBAs.

6 Training supplies included resuscitation mannequins and printed materials.

7 Additional non-training supplies included in fixed costs were all supplies provided to TBAs on a per-TBA basis: instruction cards, Laederal resuscitation masks, and plastic spoons.

8 Supply costs included among variable costs were: infant receiving blankets, bulb syringes, and amoxicillin tablets.

9 Estimated on a per-birth basis.

10 Annualized for program implementation period only (i.e., excluding set-up period but including initial training).

11 Cost per birth attended by intervention TBAs in 2006–08 program (1889) and estimated for 2011–2020 projected period (9120).

In the forecasted analysis, the estimated 2011 PV of the base case program was $256,455, with annual costs of $26,834, far lower than in the financial and economic analyses of the 2006–08 intervention, where annual costs were $49,469 and $53,550, respectively (Table 4). Lower annual cost resulted largely from spreading out first year start-up costs over a longer timeframe. Moreover, in contrast with the cost drivers for the 2006–08 program, training for the 10-year program comprised less than 50% of total costs, rather than nearly two-thirds, whereas monitoring and supervision represented almost one-third of total costs, or about double the proportion of LUNESP's costs. These differences were mainly due to task shifting from the US-based neonatologist to a local trainer (i.e., the lead Zambian trainer, a nurse-midwife). Despite savings from these adjustments, fixed costs were still projected to account for over 80% of total intervention cost. Task-shifting and compensating TBAs had minimal effect on the basic cost structure of the program. Given the low prices of variable cost items—infant receiving blankets, bulb syringes, amoxicillin tablets, and compensation to TBAs—fixed costs still far exceeded variable costs. Overall, the model predicted that the estimated cost per birth would be less than one-half that of LUNESP's economic resource cost (US$29 vs. $68). The variable cost per birth, representing the additional cost of having a trained TBA attend a delivery, was US$5.3, compared to US$8.4 for the 2006–08 trial.

The cost per neonatal death avoided and DALYs averted for both the 2006–08 program and the 2011–2020 program are summarized in Table 5. From the 33 neonatal deaths that were avoided (undiscounted) in 2006–08, we estimated that 158 deaths would be avoided in 2011–2020. For the 2006–08 program, the economic cost per death avoided was $3,900 while the cost per DALY averted was $176 (2011 US dollars). The 10-year forecasted program was much more cost-effective than LUNESP. The base case analysis predicted that the cost per death avoided was $1,866, while the cost per DALY averted was $74. ‘Optimistic’ vs. ‘conservative’ scenarios yielded values of $591 vs. $3,024 and $24 vs. $120 for cost per death avoided and DALY averted, respectively. Given that Zambia's 2011 GDP per capita was estimated to be just under $1400 (in current prices) [18], this analysis suggested that all scenarios of the intervention, including the conservative scenario, would be highly cost-effective.

**Table 5:** Program cost-effectiveness: 2006–08 and 2011–20.

**Neonatal deaths avoided**	
Neonatal deaths avoided, 2006–08 (undiscounted)	33.1
Neonatal deaths avoided, 2006–08 (discounted @ 3%)	32.8
Neonatal deaths avoided, 2011–20 (undiscounted)	157.6
Neonatal deaths avoided, 2011–20 (discounted @ 3%)	137.4
**DALYs averted** 1	
DALYs averted, 2006–08	725
DALYs averted, 2011–20	3,451
**Cost per neonatal death avoided** 2	
Financial cost per death avoided, 2006–08	3620
Economic cost per death avoided, 2006–08	3900
Estimated economic cost per death avoided, 2011–2020	
Base case	1866
Optimistic scenario	591
Conservative scenario	3024
**Cost per DALY averted** 2	
Financial cost per DALY averted, 2006–08	163
Economic cost per DALY averted, 2006–08	176
Estimated economic cost per DALY averted, 2011–2020	
Base case	74
Optimistic scenario	24
Conservative scenario	120

1 Death of a neonate = 21.9 DALYs averted, estimated by the formula given by Murray, 1994.

2 All costs expressed in real 2011 US$, with annual values discounted at 3%.

### Sensitivity analyses

The one-way sensitivity analyses showed that the 10-year base case results were most sensitive to variation in two policy-determined variables: the degree of involvement of the US-based neonatologist and number of program TBAs (Table 6). If the neonatologist attended all 3 trainings each year instead of none, the total cost per outcome (death avoided, DALY averted, and birth attended by TBA) would nearly double. Similarly, if the trainings expanded to include 100 TBAs per session rather than 60, cost-effectiveness would increase by approximately 20%. The results were also sensitive to variation in the effect size of the intervention and productivity of a given TBA (i.e., the average monthly number of deliveries attended). If the intervention reduced neonatal mortality by 8.0 (vs. 17.9 in the base case) per 1,000 live births, the cost per death avoided and DALY averted would increase by over 100%. If the intervention was implemented in an area where TBAs helped deliver more babies, e.g., 5 instead of 1–2 per month, cost-effectiveness would improve by more than 50%.

**Table 6:** Results of one-way sensitivity analysis on the incremental cost per birth attended, cost per death avoided, and cost per DALY averted of LUNESP package of neonatal interventions: 2011–2020 program (2011 US$).

Variation tested	Cost per expected birth attended by TBA	Cost per death avoided	Cost per DALY averted
Base case	$29	$1866	$74
Mortality difference (intervention impact) varied between 24/1,000 and 8/1,000 (base case = 17.9)	no change	$1392–4175	$55–166
Average number of births/month/TBA varied between 5 and 1 (base case = 1.3)	$12–36	$740–2301	$30–92
Number of participating TBAs varied between 100 and 40 (base case = 60)	$24–36	1521–2298	$61–92
Number of training workshops varied between 1 and 3 per year (base case = 3 per year)	$20–29	$1288–1866	$51–74
Number of training workshops attended by US expert varied between 0 and 3 per year (base case = 0 per year)	$29–52	$1866–3314	$74–132
Cost of food per day/participant at trainings varied +/−25%, between $8.1–13.5 (base case = 10.8)	28–31	1768–1963	70–78
Cost of travel per month for program monitoring varied +/−25%, between $356–593 (base case = 74)	27–31	1753–1978	70–79

The multivariate sensitivity analyses indicated that the results were robust to simultaneous variation in key input parameters (Table 7). The forecasted median values of cost per death avoided and DALY averted, for all scenarios, were within 5–6% of the point estimates. The 90% probability values were generally less than twice the point estimates. With 90% probability, the cost per DALY averted of the base case scenario would be less than $148, and for the conservative scenario would be less than $276. Thus, even under very conservative policy-determined conditions and extreme outcome conditions, the intervention should still be considered highly cost-effective.

**Table 7:** Multivariate sensitivity analysis of key uncertain variables and incremental cost per birth attended, cost per death avoided, and cost per DALY averted of LUNESP package of neonatal interventions: 2011–2020 neonatal survival program (2011 US$).

	Incremental cost and cost-effectiveness ratios, point estimates			Incremental cost and cost-effectiveness ratios, Monte-Carlo simulation^1^		
	Base case	Optimistic scenario	Conservative scenario	Base case	Optimistic scenario	Conservative scenario
**Cost per death avoided** 2	1866	591	3024	1960	610	3210
Minimum-maximum range				746–16683	293–3938	1106–45309
90% probability value				3706	1087	6922
**Cost per DALY averted** 2	74	24	120	76	24	128
Minimum-maximum range				27–664	12–157	44–1805
90% probability value				148	43	276
**Cost per birth attended** 2	29.1	9.2	35.4	29.2	9.2	35.5
Minimum-maximum range				18.6–76.3	7.6–16.9	20.2–232.6
90% probability value				43.3	11.5	63.5

1 20,000 trials were run for each simulation.

2 Monte-carlo simulation values are the median value.

## Discussion

This analysis indicted that training TBAs to perform interventions targeting birth asphyxia, hypothermia, and sepsis was highly cost-effective according to the WHO-accepted definition of cost-effectiveness. Furthermore, modifications to localize investments and spread them out over a 10-year program horizon would significantly enhance the cost effectiveness of the intervention. Some studies have identified even more cost-effective interventions to improve child survival in certain settings such as India ($7 per DALY averted for home-based neonatal care) [19], and in a number of countries in the same African region as Zambia ($8 per DALY averted for community newborn care) [20]. However, our results compare favorably with the range of interventions presented by the WHO as cost-effective in very low-resource environments, such as delivery by a skilled birth attendant ($37 per DALY averted) and community-based management of serious neonatal infections ($54 per DALY averted) [20].

Important drivers of our results were the intervention's effect size (reduced mortality) and high fixed costs. The latter was related to the nature of the intervention, which requires substantial training and retraining over time. However, as the sensitivity analysis and 10-year model illustrate, there are ways to task-shift and adapt the training to dramatically reduce some of these costs. By contrast, the former was entirely a function of the local population structure and the low average number of deliveries the TBAs attended—largely due to Lufwanyama's relatively sparse population density (∼6.4 persons/kilometer, roughly one third of Zambia's average [21]). Thus, the greatest economies are likely to be achieved when TBAs have the opportunity to apply their skills more frequently. In this regard, LUNESP's cost-effectiveness might underestimate the cost-effectiveness that could be achieved in a more densely populated setting.

### Strengths and weaknesses

The prospective collection of cost data during the course of an actual field study, rather than estimating costs, is an important strength of the study. Similarly, the intervention's effect size was measured directly from a methodologically-rigorous randomized controlled trial, rather than from assumptions or expert opinion. Both of these enhance the validity of our findings. The forecasted economic analysis takes the analysis one step further, creating several potential scenarios of how an intervention of this kind, conducted in a similar population but over a 10-year time scale, might perform programmatically. In addition, our sensitivity analysis identified several policy- or program-determined variables that could substantially reduce costs and increase sustainability. This provides useful information for policy makers interested in achieving the maximum yield for public health investments.

Several potential limitations of the 10-year projected intervention merit discussion. First, our results are derived from one particular setting. While they may be generalizable to similar contexts characterized by low population density and underdeveloped transportation and health systems where home births are often the only option, the precise degree to which the efficiencies we achieved in the 10-year program can be gained in other settings is uncertain. For example, the LUNESP TBAs were highly motivated, largely because of their participation in a foreign-managed project. Whether TBAs participating in a completely locally-run program would be equally eager and conscientious in applying their skills is impossible to predict. However, it could also be argued that the Lufwanyama context represented a relatively extreme situation in terms of the difficulties of introducing an intervention of this kind. The LUNESP TBAs served a vast geographic area, which made it more difficult for them to access their clients and increased the logistical complexity and costs associated with training workshops and program monitoring. The TBAs' educational levels were also low; 17% had never attended school and only 13% had progressed beyond primary school [5]. Moreover, as mentioned above, LUNESP's TBAs attended relatively few deliveries (an average of 1.2 each month), which had an adverse impact on the intervention's impact and hence lowered cost-effectiveness.

Second, we modified the trial's protocol in the 10-year program, such as the retraining frequency and the number of TBAs per training. In reality, there are limits to the changes in training one can make without decreasing intervention effectiveness. In support of this, Carlo et al, working with an unrelated group of Zambian TBAs, observed significant attrition of skills when TBAs were reassessed six months after primary training [22]. Therefore, making such changes would require a monitoring strategy to ensure that essential skills are acquired and retained. However, it is important to bear in mind that in sparsely-populated areas such as Lufwanyama, where TBAs average 1–2 deliveries monthly, skill retention is likely poorer than in settings where TBAs can attend closer to 5–6 deliveries per month. In more populous areas, the increased utilization of skills could be self-reinforcing and thus permit fewer refresher workshops. This emphasizes the need to assess TBAs' skills regularly and systematically in order to optimize retraining efforts.

Third, our results assume that task shifting from expensive and experienced US-based experts to local experts paid at local wages will not compromise the intervention's effectiveness. We consider this a reasonable assumption: neonatal resuscitation is not technically complex to teach or to perform, and should not require the very high expertise of tertiary care center neonatologist. In other settings, training skills have been transferred successfully to local trained staff such as mid-wives [23].

Fourth, we lacked the data to incorporate the treatment of conditions for children who survived due to the work of the TBAs (additional costs) as well as the treatment of perinatal complications which were averted by the involvement of the TBAs (cost savings). The incorporation of such additional potential costs and savings would inevitably strengthen our analyses.

Finally, in our forecasted model, we used a simple, linear progression of costs over time after making initial baseline cost assumptions for year 1 (2011), rather than employing more sophisticated techniques, including non-linear cost projections. However, handling the inherent uncertainty of future values with sensitivity analysis encompassing a range of scenarios and a Monte Carlo simulation analysis is a rigorous approach that is intuitive and transparent. In addition, a rigorous cost-effectiveness analysis of a nationwide version of the intervention might provide even more relevant information for policy-makers. Yet given the variation in so many key parameters that such an analysis would need to encompass, and our lack of reliable data on these varying parameters, we do not believe such an analysis would produce reliable results. We hope instead that our rigorous analysis of a modestly-sized program, based as it is on trial-generated data with transparent modifications and a careful examination of uncertain variables, can be used for programmatic purposes in settings similar to that of LUNESP.

### Conclusion and policy implications

This analysis indicates that training TBAs in practical skills targeting the key causes of neonatal mortality is highly cost-effective. Moreover, it shows that the cost-effectiveness of this strategy can likely be improved if implemented programmatically over a longer time horizon and with deliberate cost-saving measures. These results further strengthen the rationale for implementing programs similar to LUNESP in other disadvantaged communities with extremely limited access to health care.
